# Aggravation of stiff-person syndrome with ophthalmoplegia after tandospirone initiation: a case report

**DOI:** 10.3389/fimmu.2026.1749793

**Published:** 2026-04-02

**Authors:** Kaiqi Yang, Weihong Chen, Yasai Tang, Shuyi Chang, Ruohan Sun, Peiyuan Lv, Yanhong Dong

**Affiliations:** 1 Graduate School of Hebei North University, Neurology Teaching and Research Office, Zhangjiakou, Hebei, China; 2 Department of Neurology, Hebei General Hospital, Shijiazhuang, Hebei, China; 3 Department of Neurology, Hebei Provincial Key Laboratory of Cerebral Networks and Cognitive Disorders, Shijiazhuang, Hebei, China; 4 Graduate School of Hebei Medical University, Shijiazhuang, China

**Keywords:** case report, diagnosis, pathogenesis, stiff-person syndrome, treatment

## Abstract

**Background:**

Stiff-person syndrome (SPS) is a rare neuroimmune disorder that is associated with a variety of symptoms and varying degrees of disability. SPS has multiple phenotypes and different immunological bases, and the lack of clinical awareness of these various clinical phenotypes often leads to misdiagnosis and inappropriate treatment of SPS patients in the early stage of the disease course.

**Case description:**

We report a distinctive case of SPS with a 4-year follow-up. The patient was a 56-year-old middle-aged woman who initially presented with diplopia and atypical muscle tension. Treatment with baclofen and lorazepam showed poor efficacy, and she was once misdiagnosed with anxiety and depression as well as oculomotor nerve palsy. During the 4-year follow-up period, the patient gradually developed typical clinical manifestations and signs of SPS and was diagnosed with positive GAD antibodies. In the later stage, the patient’s symptoms were exacerbated by the serotonin receptor partial agonist tandospirone; further testing revealed a significant increase in GAD antibody titer.

**Results:**

As the patient could not tolerate hormone pulse therapy, she was administered rituximab and two sessions of intravenous immunoglobulin (IVIG). After adjustment of the relevant treatment regimen, the patient’s symptoms were significantly alleviated.

**Conclusion:**

SPS is relatively uncommon and prone to misdiagnosis. Therefore, we need to understand the various clinical manifestations of SPS, achieve early intervention, and thereby improve the prognosis and quality of life of patients.

## 1 Introduction

SPS is an autoimmune disease primarily characterized by chronic progressive muscle rigidity. It presents with stiffness in the limb and axial muscles, stiff gait, and uncontrolled fall episodes, as well as paroxysmal, painful muscle spasms triggered by anxiety, fear in specific environments, and startle responses (1). Clinical symptoms can be well controlled with standardized treatment. In this article, we present a case study of stiff-person syndrome (SPS) with a 4-year follow-up, in which the patient’s condition was exacerbated by tandospirone, aiming to enhance our understanding of this disease.

## 2 Clinical information

### 2.1 Initial presentation (2020)

In January 2020, a 56-year-old female patient presented to our hospital due to left oculomotor nerve palsy. She first experienced vertical diplopia (double vision) and inability to focus her vision, especially when viewing objects at night, she clearly felt a narrowed visual field and constricted visual space, and worried about falling due to blurred vision. Physical examination revealed left ptosis and limited upward gaze of the left eye, and the diagnosis of left oculomotor nerve palsy was made at that time. Symptomatic treatment with eperisone was given, but the patient reported no significant improvement in her symptoms.

### 2.2 Diagnosis and stabilization (2021-2024)

**Figure 1:**
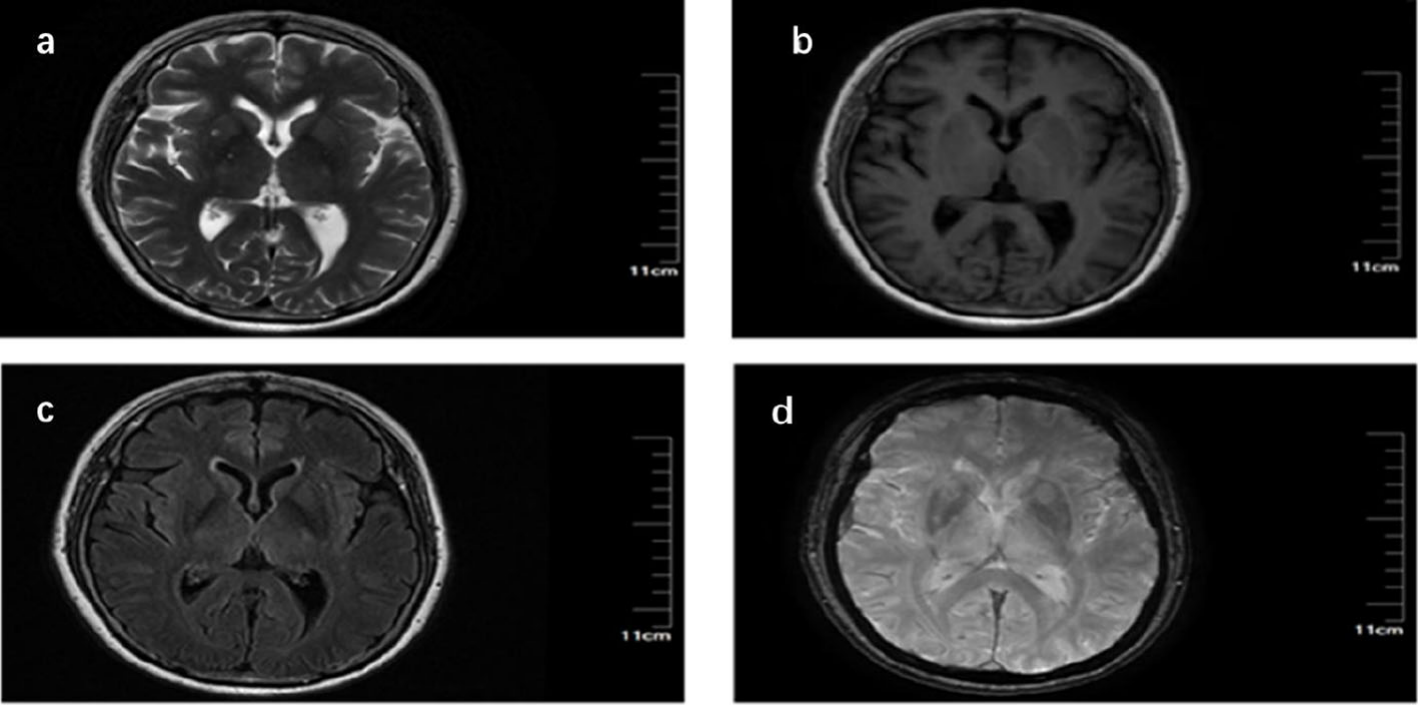
Magnetic Resonance Imaging (MRI) and Susceptibility-Weighted Imaging (SWI). **(a)** T2-weighted imaging (T2WI) shows small patchy hyperintensities in the bilateral frontal and parietal lobes, and a small patchy long T2 signal in the right basal ganglia region; **(b)** T1-weighted imaging (T1WI) reveals a long T1 signal in the right basal ganglia region; **(c)** Fluid-attenuated inversion recovery (FLAIR) imaging demonstrates a signal with hypointensity in the center and hyperintensity in the periphery in the right basal ganglia region, as well as rim-like hyperintensities around the bilateral lateral ventricles; **(d)** Susceptibility-weighted imaging (SWI) shows no abnormal hypointensities.

In August 2021, the patient developed abdominal muscle rigidity and reported feeling stiff abdominal muscles, unsteady gait when walking, and fear of falling. She stated that her symptoms worsened when she was emotionally agitated or irritable, and she felt limited eye movement. Re-examination revealed mild abdominal muscle rigidity, bilateral ptosis, limited upward and downward gaze of both eyes, and limited adduction of both eyes; there was no gait or activity limitation, and no muscle spasm. Treatment with baclofen and lorazepam yielded poor results. Further imaging examinations indicated lacunar infarction in the right basal ganglia and chronic ischemic cerebral changes (**Figure 1**). Neuropsychological scales suggested an anxiety-depressive state, but treatment with sertraline had little effect, and the symptoms continued to progress. The patient gradually developed an unsteady gait and a wide-based gait. Electromyography (EMG) showed continuous action potential discharge in the right rectus abdominis muscle and left orbicularis oculi muscle during the resting phase (**Table 1**). Cerebrospinal fluid (CSF) testing (sent to an external laboratory) showed positive GAD65 antibody (titer 1:100), and blood testing also showed positive GAD65 antibody (titer 1:32) (tested at Peking Union Medical College Hospital Laboratory); paraneoplastic antibodies were negative. The patient had a past medical history of Sjögren’s syndrome (positive for RO-52 antibody), subclinical hypothyroidism, and iron deficiency anemia. After treatment with oral estazolam, clonazepam combined with intravenous immunoglobulin (IVIG), the patient’s symptoms improved significantly; over the subsequent 4 years, the patient regularly took estazolam, clonazepam and methylprednisolone outside the hospital, reported being able to live a normal life, felt significant relief of muscle rigidity compared with before, and could walk normally.

**Table 1:** The electromyography (EMG) results of this patient are as shown above.

Date	Side	Muscle	Insertional	Fibs	Wave	Fase	Amp(uv)	Dur(ms)	Poly(%)	Amp(mv)	Pattern	Effort
2021/8/20	Right	Rectus abdominis	(-)	(-)	(-)	(-)	/	/	/	/	/	Recontraction
	Right	Gastrocnemius muscle	(-)	(-)	(-)	(-)	1052	10.77	0	2.0	Interfering phase	Recontraction
	Left	Orbicularis oculi muscle	(-)	(-)	(-)	(-)	/	/	/	/	/	Recontraction
2021/9/7	Right	Rectus abdominis	(-)	(-)	(-)	(-)	/	/	/	/	/	Recontraction

• On August 20, 2021, Continuous action potential discharge was observed in the right rectus abdominis muscle and left orbicularis oculi muscle during the resting phase.

• On September 7, 2021, A follow-up electromyography (EMG) following oral administration of estazolam and clonazepam plus intravenous human immunoglobulin (IVIG) revealed continuous action potential discharges in the right rectus abdominis muscle, with a reduction compared to the prior(August 20)findings.

• “(-) or/” indicates no abnormal findings for the corresponding examination.

### 2.3 Aggravation event with tandospirone (2025)

**Figure 2:**
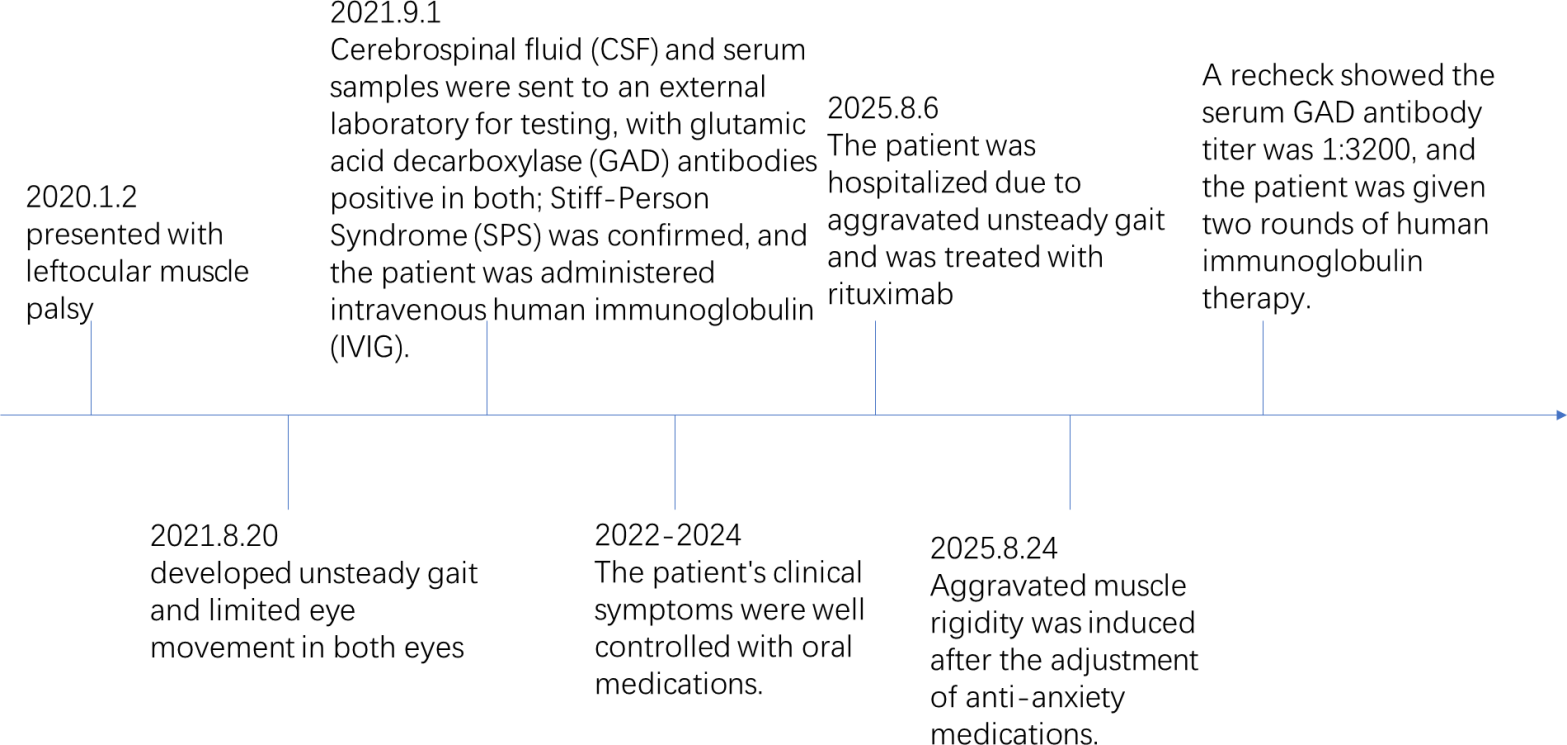
The patient’s disease progression and treatment process.

**Figure 3:**
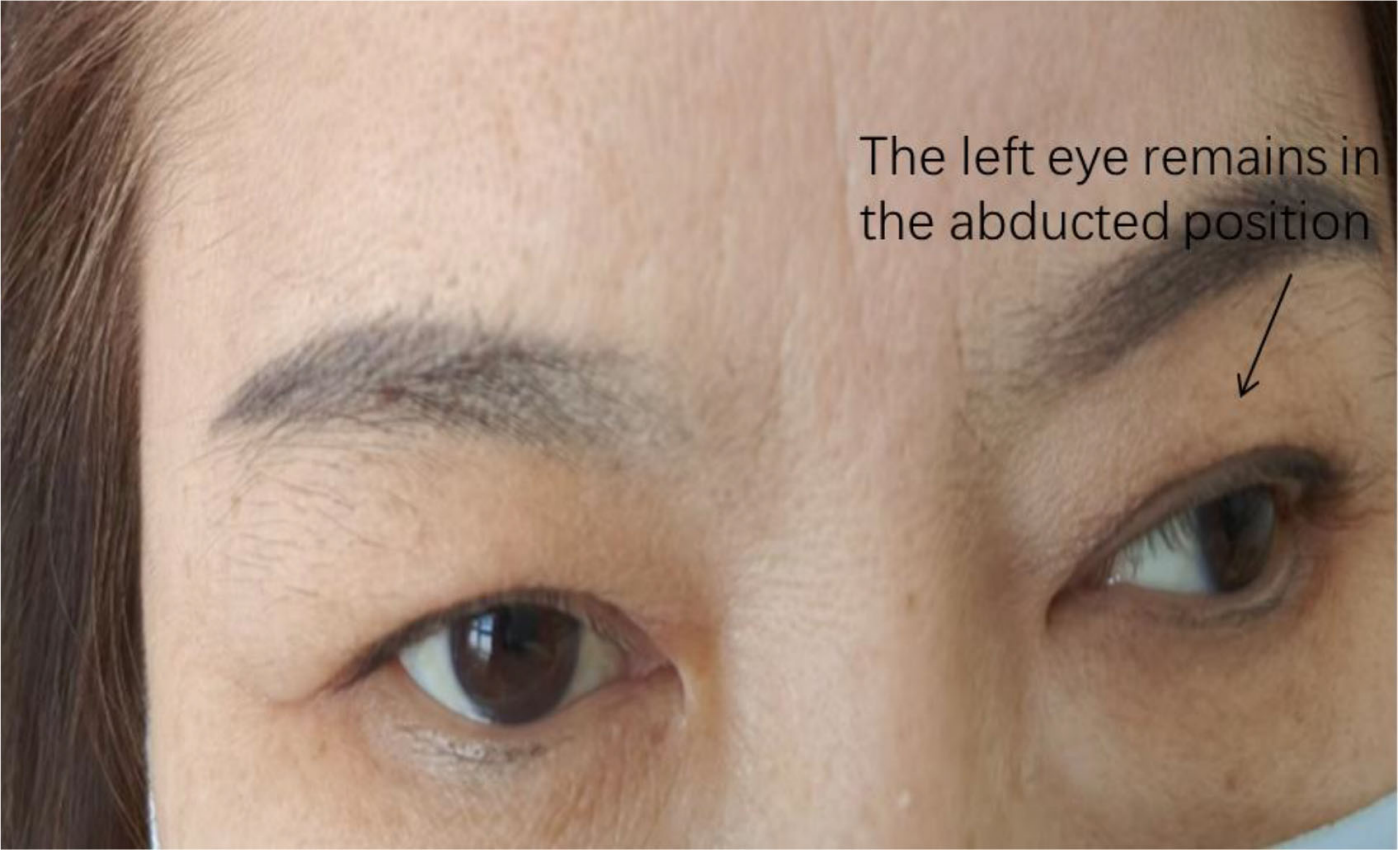
The left eye remains in the abducted position when gazing straight ahead.

In August 2025, the patient reported significant emotional fluctuations, so tandospirone was added. Subsequently, the patient stated that her abdominal muscle rigidity worsened, accompanied by convulsive seizures, which were induced by audio-visual and light stimuli, leading to multiple falls. She reported being unable to stand or walk independently during seizures, so she was re-admitted to the hospital for treatment. Physical examination during re-admission showed abdominal muscle rigidity and board-like abdomen; transient and severe convulsions, lasting several seconds to minutes and accompanied by unbearable pain and discomfort, could be induced by sudden sounds, touch, light changes or voluntary movements (such as attempting to walk, turn over, bend over, etc.). Re-examination showed that the serum titer of GAD65 antibody was 1:3200 (tested at Chengdu Haimoyunyin Medical Laboratory). It should be noted that the two laboratories adopted the same detection method for GAD antibodies. Unfortunately, after admission during this hospitalization, the patient was unable to cooperate with the electromyography (EMG) examination due to physical reasons. After treatment with rituximab (500 mg), the patient reported no improvement in abdominal muscle rigidity and unsteady gait and developed secondary COVID-19 infection after the use of immunosuppressants. Suspecting that the administration of tandospirone exacerbated the patient’s clinical symptoms, the drug was discontinued immediately. After receiving two courses of intravenous immunoglobulin (IVIG) pulse therapy (0.4 g/kg/day), combined with methylprednisolone 40 mg once daily (gradually tapered to 20 mg once daily for oral administration) and dosage adjustments of estazolam and clonazepam (**Figure 2**), the patient’s symptoms improved significantly. She reported being able to walk normally, with no recurrence of convulsions; she felt that her ptosis and eye movement function had improved significantly compared with before, but limited adduction of the left eye persisted (**Figure 3**). However, the patient reported that her psychological problems had been significantly relieved compared with before, and she could live a normal life.

## 3 Discussion

Stiff-person syndrome (SPS) was first reported and named by Moersch and Woltman in 1956 (2), and its pathogenesis remains unclear. Autoantibodies against glutamic acid decarboxylase (GAD) are detected in 70% to 80% of patients. GAD, a key intracellular enzyme responsible for the synthesis of the inhibitory neurotransmitter γ-aminobutyric acid (GABA), exists in two isoforms, GAD65 and GAD67, in the mammalian brain: GAD67 binds to the pyridoxal phosphate (PLP) cofactor and mediates the basal synthesis of GABA (3), while GAD65 is PLP-independent and enables the rapid synthesis of GABA (4). Anti-GAD antibodies in SPS patients primarily inhibit GAD65; they block GABA synthesis by reducing the uptake and release of newly synthesized GABA into synaptic vesicles, thereby impairing GABAergic transmission and triggering neuronal hyperexcitability (5). This constitutes the core pathophysiological mechanism of SPS: the reduced firing of inhibitory GABAergic neurons in the spinal cord leads to hyperexcitability of motor neurons and synchronous contraction of agonist and antagonist muscles. Recent studies have confirmed that the autoimmune mechanism of SPS is also associated with T cell activation: infiltration of CD8+ and CD4+ T cells has been observed in the spinal cord and brainstem of deceased SPS patients (6), and GAD65-reactive CD4+ T cells can induce fatal encephalomyelitis in mice, with their numbers and the depth of parenchymal brain infiltration increasing as the disease progresses (7).

The incidence of SPS is estimated to be 1–2 cases per million population, with a higher prevalence in females, and the typical age of onset is 30–50 years. It can be complicated by other autoimmune diseases, including autoimmune thyroid disease, Sjögren’s syndrome and systemic lupus erythematosus (8). The clinical and auxiliary examination findings of the present patient were consistent with the diagnostic criteria for classic SPS (1), whose core features included muscular involvement of the trunk and limbs, positive glutamic acid decarboxylase (GAD) antibodies, and muscle spasms triggered by precipitating factors. Furthermore, the patient was comorbid with subclinical hypothyroidism and Sjögren’s syndrome, which provided more robust evidence for the diagnosis in this case. With regard to auxiliary examinations, no obvious abnormalities were identified on cranial MRI, and paraneoplastic antibodies were negative, ruling out organic intracranial lesions and paraneoplastic etiologies. Electromyography (EMG) revealed continuous action potential discharges at rest in the right rectus abdominis and left orbicularis oculi muscles; combined with a marked elevation in the rechecked GAD antibody titer, these findings further supported the immune-mediated pathogenesis of SPS. In addition, the patient’s muscle rigidity exacerbated following the addition of tandospirone, and muscle spasms could be induced by external stimuli such as tactile, electrical, auditory or visual stimulation. Moreover, the patient exhibited a favorable therapeutic response to benzodiazepines (estazolam and clonazepam), as well as rituximab and intravenous human immunoglobulin (IVIG). These clinical manifestations and treatment responses were highly consistent with the clinical diagnostic and therapeutic characteristics of SPS, which further confirmed the accuracy of the diagnosis.

Patients with stiff-person syndrome (SPS) may present with abnormal eye movements. Economides JR. et al. reported an SPS patient without comorbidities such as myasthenia gravis or Graves’ disease, who developed right eye strabismus, saccadic initiation dysfunction, and nystagmus (9). Meanwhile, Oskarsson et al. also reported a case of SPS complicated with vertical gaze palsy; after administration of human immunoglobulin, the patient’s eye movement function and limb stiffness improved significantly (10). These findings indicate that there is increasing evidence that eye movement disorders can occur in SPS patients. The present patient’s initial symptom was limited upward gaze of the left eye, followed by impaired eye movement in all directions of both eyes, and significant improvement was observed after treatment with human immunoglobulin. The underlying mechanism is considered to be continuous discharge of oculomotor nerve neurons caused by GABA dysfunction.

After the patient was administered tandospirone, their muscle rigidity aggravated. Tandospirone belongs to the class of 5-hydroxytryptamine (5-HT) receptor agonists (serotonin receptor partial agonists, S-RPA) and exerts an anxiolytic effect by acting on 5-HT1A receptors in the brain to inhibit 5-HTergic neural activity. Sablaban et al. reported a case where the use of buspirone led to exacerbated panic attacks and generalized spasms. After ruling out serotonin syndrome and neuroleptic malignant syndrome, the exacerbation of SPS symptoms was attributed to buspirone (11). 1-(2-Pyrimidyl)piperazine (1-PP) is the major *in vivo* metabolite of buspirone, and this metabolite is also a common metabolite of other antidepressant/anxiolytic drugs that act as 5-HT1A receptor agonists, including tandospirone. Studies have shown that low doses of 1-(2-pyrimidinyl)piperazine (1-PP), a common metabolite of 5-HT1A agonists, can reverse the inhibitory effect of α2-adrenergic receptor agonists on the firing activity of locus coeruleus norepinephrine (NE) neurons (12). A possible mechanism can also be explained by the observation that buspirone increases the activity of locus coeruleus noradrenergic neurons *in vitro*. Previous case reports have clearly demonstrated that the use of selective serotonin reuptake inhibitors (SSRIs) can result in the exacerbation of SPS symptoms (13, 14). This patient had never been treated with SSRIs such as citalopram, which further confirms that 5-HT receptor agonists can also induce the exacerbation of SPS symptoms. Therefore, when treating anxiety symptoms in patients with SPS, we should use medications cautiously and consider all relevant factors comprehensively.

The treatment of SPS mainly includes pharmacotherapy (symptomatic and immunotherapeutic drugs) and non-pharmacological treatment. In symptomatic treatment, benzodiazepines are the cornerstone of therapy. As GABA_a_ agonists, they can relieve muscle spasms and also alleviate symptoms of anxiety and depression. The patient’s symptoms improved significantly after oral administration of clonazepam, estazolam, and other benzodiazepines during symptom attacks (15). α_2_-adrenergic agonists (such as tizanidine and clonidine) can also be used for symptomatic treatment of SPS. Salvatore et al. reported a case of clonidine being used in the treatment of SPS crisis, indicating that clonidine can serve as an adjuvant therapy for acute exacerbation of SPS or SPS crisis; however, adverse reactions such as hypotension and somnolence should be cautiously monitored (16). Non-pharmacological treatments, including stretching exercises, ultrasound therapy, gait and balance training, acupuncture, and physical therapy, can also relieve the clinical symptoms of SPS. Targeting the autoimmune mechanism in SPS, immunotherapy can be initiated, which mainly includes glucocorticoids, intravenous immunoglobulin (IVIG), and plasma exchange. IVIG is the first-line immunotherapy. It can significantly improve limb and muscle stiffness in patients and reduce anxiety-induced spasms. For patients with impaired venous access or early fading of therapeutic effects, subcutaneous immunoglobulin injection can be adopted (15). For patients with poor response or non-response to IVIG, rituximab (RTX) can be selected. RTX is a chimeric monoclonal antibody targeting the CD20 antigen; studies have shown that RTX does not interfere with the production of immunoglobulins by plasma cells or the regeneration of B cells from progenitor cells. A systematic review on RTX for SPS treatment indicated that RTX is generally well-tolerated with few side effects. Most patients achieved significant clinical improvement, and a small subset of patients achieved complete remission (17). Anti-CD19 chimeric antigen receptor T-cell therapy (CAR-T therapy) has been proven effective in the treatment of refractory SPS, with a significant decrease in antibody titer and obvious improvement in limb stiffness observed (18). Other immunotherapeutic approaches also include plasma exchange and autologous hematopoietic stem cell transplantation.

## 4 Patient perspective

At the initial stage of the disease, I experienced double vision, with obvious vertical diplopia. In the evening, objects appeared thinner and narrower to me. Fearing instability and falls, I initially thought the problem was only with my eyes. However, my symptoms did not improve significantly after taking related medications. Subsequently, I gradually developed stiffness and tightness in the abdominal muscles, accompanied by unsteady and staggering gait. I was constantly worried about falling, and these symptoms were significantly aggravated by tension and anxiety. After being diagnosed with stiff-person syndrome, I experienced certain psychological stress, but I remained positive and actively cooperated with all treatments. During disease relapse, my mood fluctuated markedly. Minor stimuli, such as the sound of a door closing, could trigger severe fear. In severe episodes, I was unable to walk normally and fell repeatedly. After active and standardized treatment, the abdominal stiffness was significantly relieved, and the muscles became much softer. My gait gradually stabilized, and I was eventually able to walk independently without assistance. My overall emotional state also improved considerably.

## 5 Strengths and limitations

The advantage of this case is that it further supplements the clinical symptom spectrum of stiff-person syndrome (SPS), further clarifies that SPS patients may be complicated with ophthalmoplegia, enriches clinicians’ understanding of the atypical symptoms of SPS, and provides valuable clinical case support for the early identification and accurate diagnosis of such atypical cases in clinical practice. Meanwhile, this case has confirmed through practice that SRPA-type anti-anxiety and antidepressant drugs may exacerbate the clinical symptoms of SPS patients, while intravenous immunoglobulin (IVIG) treatment has definite efficacy in SPS patients, providing a direct reference basis for the clinical medication decision-making of SPS patients. Based on the findings of this case, the future direction of clinical diagnosis and treatment can focus on two aspects: first, to strengthen the attention and summary of the atypical symptoms of SPS, and improve the clinical diagnosis system of SPS; second, to further explore the standardized rational selection of anti-anxiety and antidepressant drugs for SPS patients, optimize the clinical regimen of IVIG in the treatment of SPS, and simultaneously expand the sample size of cases to carry out relevant studies to verify the efficacy and safety of medication, to provide a more solid clinical basis for the individualized diagnosis and treatment of SPS patients and improve the prognosis of patients.

## Data Availability

The original contributions presented in the study are included in the article/Supplementary Material. Further inquiries can be directed to the corresponding author.
